# Organizational Learning and Primary Care Nurses' Work Performance and Well-Being: A Multilevel Linear Analysis in a Developing Country

**DOI:** 10.1155/2024/2770347

**Published:** 2024-07-16

**Authors:** Ruixue Zhao, Wenhua Wang, Jinnan Zhang, Mengyao Li, Stephen Nicholas, Elizabeth Maitland, Huiyun Yang, Rebecca Mitchell

**Affiliations:** ^1^ School of Public Policy and Administration Xi'an Jiaotong University, Xi'an, China; ^2^ Australian National Institute of Management and Commerce, Sydney, Australia; ^3^ Health Services Research and Workforce Innovation Centre Newcastle Business School University of Newcastle, Newcastle, Australia; ^4^ School of Management University of Liverpool, Liverpool, UK; ^5^ Health and Wellbeing Research Unit (HoWRU) Macquarie Business School Macquarie University, Sydney, Australia

## Abstract

**Aim:**

This study aims to investigate the level of organizational learning within urban Chinese Community Health Centres and reveal its potential association with primary care nurses' work performance and well-being.

**Background:**

Globally, there is a push to establish learning healthcare systems for complex health reform challenges. Existing studies on organizational learning mainly focus on North American and European hospital settings, offering limited insights into primary care environments, particularly in developing countries.

**Design:**

Cross-sectional study.

**Methods:**

We recruited 175 nurses from 38 community health centres in four Chinese cities (Shanghai, Shenzhen, Tianjin, and Jinan) using convenience sampling. Trained research assistants conducted face-to-face surveys, measuring organizational learning with the Learning Orientation Scale. Nurse-level outcomes included self-directed learning, quality of care, organizational commitment, and work stress. Data analysis employed multilevel linear modelling.

**Results:**

The 38 community health centres displayed a relatively high level of organizational learning, and there was a positive and significant association between organizational learning within community health centres and nurses' self-directed learning as well as the quality of care. However, there was no significant association between organizational learning and nurses' organizational commitment or work stress.

**Conclusion:**

This study demonstrates a high-level organizational learning capacity in urban community health centres in China. It provides a new perspective on the potential relationship between CHCs' organizational learning and primary care nurses' well-being and work performance. Further research is needed to clarify unexpected findings and identify factors promoting organizational learning in primary care settings. *Implications for Nursing Management*. In China's evolving primary care system, nurses play a vital role amidst physician shortages. Policy should prioritize internal management reform alongside structural changes. This study highlights the importance of fostering organizational learning in primary care settings. Strategic interventions should promote a learning culture in CHCs, which may enhance nurses' self-directed learning and improve the quality of care.

## 1. Introduction

Today's healthcare systems operate in an uncertain, turbulent, and interconnected environment, facing constant pressure from policymakers, healthcare analysts, and funders to provide quality healthcare while also maintaining operational efficiency and cost-effectiveness [1]. Internationally, there are reform initiatives aimed at establishing high-performing primary care delivery models, such as the Patient-Centred Medical Homes (PCMHs) and Accountable Care Organizations (ACOs) in the United States [2], Family Health Teams and Family Medicine Groups in Canada [3], and Family Doctor Teams in China [4].

The ever-shifting demographics, evolving political priorities, and ongoing financial concerns continually introduce new challenges into the healthcare landscape, meaning that these current reform initiatives have a finite lifespan. One healthcare constant is the system's ability to adapt and respond effectively to these changing conditions and evolving patient needs [5]. This adaptation requires ongoing experimentation and a commitment to change, reliant on the internal capacity for organizational learning and development [6]. For example, a recent study in the UK examined how a large healthcare organization responded to complex and disruptive changes, notably during the recent pandemic. The study revealed that the organization transformed into a learning organization, which is a critical factor for effectively addressing disruptive changes and facilitating postpandemic organizational recovery [7].

Scholars hold diverse perspectives on organizational learning. Some argue that organizational learning takes place when members of an organization act as learning agents, responding to changes in the organization's internal and external environments by detecting and correcting errors in the organization's theory in use [8]. The concept of organizational learning also can be traced back to the idea of transferring learning from one person to another, as put forth by Taylorism, with the aim of enhancing organizational efficiency, development, and performance. This dynamic process enables employees to swiftly adapt to changes and promotes the development of new behaviours and skills within the organization [9].

In the healthcare sector, prominent institutions such as the National Academy of Medicine (NAM), the National Institutes of Health (NIH), and the Agency for Healthcare Research and Quality (AHRQ) are advocating for healthcare organizations to transform into “learning healthcare systems” (LHSs). The LHS concept emphasizes the need for healthcare organizations to adopt a more systematic and data-driven approach to generate and leverage knowledge, ultimately improving the quality and value of the care they provide while fostering innovation [10].

Proficiency in organizational learning is imperative for attaining a high level of reliability in a dynamic healthcare environment [11]. Through the practice of organizational learning, healthcare teams can consistently deliver safe, efficient, and high-quality care in the midst of a complex and ever-evolving environment [12]. Nurses, the largest group involved in healthcare delivery, hold a critical role in establishing caring and therapeutic relationships with patients [13]. They serve as pivotal sources for knowledge transfer within healthcare organizations, making them instrumental in the process of organizational learning [13]. In China, primary care nurses are increasingly assuming healthcare responsibilities that extend beyond their traditional roles. Nurses assist physicians, provide clinical follow-up care and home nursing, and manage patients' electronic health records. This expanded role is particularly crucial in the context of an aging population, the growing prevalence of chronic conditions and mental health issues, and new challenges like those posed by the COVID-19 pandemic [14]. Organizational learning is arguably important for the effective management of nurses during periods of transition [15].

Within the healthcare literature, researchers have extensively examined the antecedents, contextual factors, and mechanisms of organizational learning across multiple dimensions, including individuals, organizations, and the environment [16–18]. However, there has been relatively limited exploration of organizational learning outcomes [19]. Most existing studies focusing on the outcomes of organizational learning have centred around the hospitals [20, 21]. There is a dearth of information regarding organizational learning and its associated outcomes within primary care settings, particularly in developing countries such as China [22]. Furthermore, there is minimal information about the influence of organizational learning on primary care nurses' work performance and well-being in China.

This study bridges the existing knowledge gap by shedding light on the level of organizational learning within community health centres (CHCs) in urban China, a vital component of the country's primary care landscape. Furthermore, it analyses the relationship between organizational learning within CHCs and the well-being and work performance of primary care nurses as they adapted to change healthcare demands. These new insights inform policies and strategies aimed at enhancing the resilience and effectiveness of China's primary healthcare delivery in the face of dynamic challenges.

## 2. Methods

### 2.1. Study Design

This study conducted a cross-sectional, in-person survey of nurses working in CHCs in China from November 2020 to May 2021.

### 2.2. Study Setting and Sampling

This study gathered data from Shanghai, Shenzhen, Tianjin, and Jinan, where well-established primary care systems boast enhanced infrastructure and a cadre of primary care professionals. This information could prove valuable in steering further reforms in primary care across these cities and regions in China. Given the variations in practice size and patient volumes in the four cities, a convenience sampling approach was employed, resulting in 38 selected CHCs with 10 in Shanghai, 14 in Shenzhen, 8 in Tianjin, and 6 in Jinan [14]. For each CHC, all nurses on duty on the day when the researchers arrived were invited to participate in the survey. A total of 175 primary care nurses from 38 CHCs, with an average of 4-5 nurses per CHC, completed the nurse survey. The survey gathered information on the nurses' personal characteristics, self-directed learning, self-reported quality of care, organizational commitment, and work stress. Data were also collected from the directors of each CHC through a primary care organization survey, focusing on organizational learning and organizational characteristics.

To ensure data quality and reliability, stringent quality control measures were implemented at various stages of the survey. Initially, experts meticulously refined the questionnaire for clarity and precision, ensuring logical consistency throughout. A presurvey was conducted to identify and address any areas for improvement. During the data collection phase, surveyors underwent comprehensive training, while nurses were briefed on the questionnaire. Real-time support was provided to nurses, and on-site checks were carried out to verify the accuracy and completeness of the responses. Following the survey, a double-entry data entry method was employed to minimize errors, followed by thorough data cleaning procedures to enhance accuracy and ensure quality assurance.

### 2.3. Measures and Variables

#### 2.3.1. Organization-Level Variables

Learning orientation can be considered the wellspring of core organizational values that influence the organization's propensity to generate and apply knowledge [8]. It plays a pivotal role in shaping the organization's satisfaction with its current operational theories, thereby affecting the extent of proactive learning [8]. A stronger learning orientation indicates a greater willingness and capacity for the organization to learn [23, 24]. Three organizational values regularly associated with the organization's inclination to learn are commitment to learn, open-mindedness, and shared vision [8].

Commitment to learn refers to the fundamental value held by an organization towards learning, representing the dedication and emphasis on fostering a learning culture. Open-mindedness refers to the organization's willingness and ability to question and challenge existing mental models, beliefs, assumptions, and routines. Shared vision entails a common and collectively agreed-upon direction or goal that guides the organization's learning and actions, providing a clear sense of purpose.

In this study, we adopted the Learning Orientation Scale developed by Sinkula et al. [8], with certain modifications to better suit the environment of primary care in China. Subsequently, we assessed organizational learning within 38 CHCs. Measured using a 5-point Likert scale (ranging from 1 “strongly agree” to 5 “strongly disagree”), specific items for each aspect are detailed in Table 1.

#### 2.3.2. Nurse-Level Variables

As shown in Table 2, our measure of self-directed learning utilized three items from the study of Yang et al. [25], self-directed learning scale among nurses. Based on the previous research studies [26], the quality of care reported by nurses was assessed using the question provided in Table 2.

Drawing on the Allen and Meyer organizational commitment model, these were designated as “affective,” “continuance,” and “normative” commitment. Employees with strong affective commitment remain with the organization because they genuinely want to; those with strong continuance commitment stay because they feel a need to do so; individuals with strong normative commitment remain committed because they feel a sense of moral or ethical obligation to stay [27]. Our modified and contextualized organizational commitment scale was based on Allen and Meyer [27], with six positively worded items listed in Table 2.

Work stress variables in Table 2 are based on Cavanaugh's concepts of challenge stressors and hindrance stressors [28]. Challenge stress refers to a series of stressors that positively influence employees' skill enhancement and career development, while hindrance stress refers to stressors that negatively affect employee career development and personal growth [29].

#### 2.3.3. Control Variables

As shown in Table 3, control variables were collected, including nurse-level variables such as age, sex, education level, and years of working experience. In addition, organization-level control variables were gathered, consisting of types of organization ownership (government-managed CHCs and hospital-managed CHCs), accreditation status (accredited by national or provincial health authorities or not), and organization size (number of staff).

### 2.4. Ethical Considerations

The study was approved by the Biomedical Ethics Committee of the Medical Department of Xi'an Jiaotong University (No. 2020-1344). All methods that we used adhered to the accepted guidelines for ethical reporting.

### 2.5. Statistical Analysis

The analytic approach involved several stages. First, Cronbach's alpha coefficients were employed to assess the internal consistency of the scales. Descriptive statistics were then utilized to provide an overview of the sample characteristics and the main variables. The Spearman rank correlation coefficient was employed to explore the relationship between organizational learning and the four nurse-level variables. Subsequently, with nurse-level data nested within the organization-level data, we constructed four 2-level hierarchical linear regression models (nurses at level 1, nested within the CHCs at level 2) to investigate whether organizational learning of CHCs influence nurse-level variables [30]. The data were analysed using Stata 15/SE.

## 3. Results

### 3.1. Characteristics of the Participants

Table 3 presents the descriptive statistics on 175 nurses. The average age of nurses is 34.93 years, ranging from 22 to 61 years; most nurses were female, accounting for 98.29% of the sample, and 93.10% of nurses had attended junior college or college. Nurses had an average of 11 years of work experience. Their tenure ranged from 0.2 to 40 years. Among the CHCs, 60.53% were government-managed and 39.47% were under the management of public hospitals. In addition, 57.89% of CHCs were accredited by national or provincial health authorities. Furthermore, 34.21% of CHCs had 35 or fewer employees, while 28.95% had more than 100 employees.

### 3.2. Reliability and Scores of Variables

Table 4 displays Cronbach's alpha for each variable scale, along with the mean and standard deviations of the variables. These variables include organizational learning (composed of three first-order factors) and four nurse-level dependent variables.

Reliability refers to the consistency and stability of results obtained when using the same measurement tool. In this study, the reliability of the measurement tools for variables was assessed using Cronbach's alpha. A higher Cronbach's alpha value indicates greater reliability of the scale. The research study suggests that a Cronbach's alpha value greater than 0.9 indicates very good reliability, while 0.7< Cronbach's alpha <0.9 indicates good reliability. Cronbach's alpha of the Learning Orientation Scale was 0.88 (with commitment learning at 0.74, shared vision at 0.81, and open-mindedness at 0.72), the Self-Directed Learning Scale was 0.83, the Organizational Commitment Scale was 0.94, and the Work Stress Scale was 0.85. These Cronbach's alpha values collectively indicate a high level of internal consistency among the items and suggest that the measurement tools used in this study are reliable, providing consistent results across the variables assessed.

The average organizational learning score for the 38 CHCs was 4.09 out of 5, indicating a high level of organizational learning. Specifically, the mean score for commitment learning was 4.25, 4.07 for shared vision and 3.96 for open-mindedness out of 5. There were differences between the three components that constitute organizational learning: the average score for self-directed learning was 4.16 (SD 0.72), quality of care was 3.17 (SD 0.54), organizational commitment was 4.04 (SD 0.88), and work stress was 3.39 (SD 0.96).

### 3.3. Correlation Analysis

Table 5 presents the correlation matrix for the main variables. The findings reveal that organizational learning exhibits positive correlations with self-directed learning (*r* = 0.179, *P* < 0.05), quality of care (*r* = 0.145, *P* < 0.10), and organizational commitment (*r* = 0.149, *P* < 0.05). However, no significant correlations were observed between organizational learning and work stress. These results suggest that higher levels of organizational learning were associated with increased self-directed learning, improved quality of care, and greater organizational commitment among the nurses.

### 3.4. Multilevel Linear Analysis

Reliability diagnosis of the multilevel ordinal logit model was conducted by calculating the intraclass correlation coefficient (ICC), which ranges from 0 to 1, to ensure the model's suitability for our analysis. If the ICC value exceeded 0.059, it indicated the necessity of performing a multilevel model for data analysis. The intraclass correlation coefficient (ICC) shows clustering effects, with the ICC values of 0.123 for self-directed learning, 0.060 for quality of care, 0.219 for organizational commitment, and 0.094 for work stress. These ICC values suggest that a significant portion of the variability in the dependent variables can be attributed to differences between CHCs. Using multilevel linear models is an appropriate approach to examine how the nurse characteristics are influenced, not only at the individual level but also at the CHC level, variables.

Table 6 presents the results of the multilevel linear models examining the relationship of organizational learning on nurse characteristics in CHCs. The key organizational variable, organizational learning, shows a positive and significant association with self-directed learning (*β* = 0.272, *P* < 0.05) and quality of care (*β* = 0.234, *P* < 0.05). The association with organizational commitment was not statistically significant (*β* = 0.265, *P*=0.146), and there was a negative but nonsignificant association with work stress (*β* = −0.226, *P*=0.175). Among the control variables, none of the nurse-level variables (age, sex, education level, and years of working experience) show statistically significant associations with any of the dependent variables (self-directed learning, quality of care, organizational commitment, and work stress). Regarding organizational level variables, organizational ownership and accreditation do not have statistically significant associations with the dependent variables. However, organizational size showed a significant association with self-directed learning (36–55 employees: *β* = 0.366, *P* < 0.05).

The Akaike Information Criterion (AIC) values were computed for both the full models and the null models, which included only the intercept. The AIC value for the full model, including both organizational learning and control variables, is 375.851, while the AIC for the model considering only self-directed learning is 377.013. Similarly, the AIC value for the full model, including both organizational learning and control variables, is 287.867, whereas the AIC for the model including only quality of care is 290.889. Notably, the AIC values of the full models were lower than those of the null models, indicating that the full models provided a better fit.

On the contrary, the AIC value of the null model containing only organizational commitment (AIC: 444.730) is lower than that of the full model including organizational learning and control variables (AIC: 448.611). Similarly, the AIC value of the null model containing only work stress (AIC: 477.673) is also lower than that of the full model (AIC: 480.625). These findings suggest that incorporating organizational learning and other control variables did not enhance the fit of these models; instead, it worsened. This suggests that the variable of organizational learning may not significantly improve the explanatory power of these models.

Table 7 presents the results of the multilevel linear models examining the three first-order factors of organizational learning and their associations with nurse characteristics in CHCs. Commitment to learn shows positive and significant associations with self-directed learning (*β* = 0.229, *P* < 0.05) and self-reported quality of care (*β* = 0.178, *P* < 0.05), while it exhibits a positive, but not statistically significant, association with organizational commitment (*β* = 0.207, *P*=0.188). There was a significant negative correlation between commitment to learn and nurses' work stress (*β* = −0.326, *P* < 0.05), suggesting that higher organizational commitment to learn is related to lower perceived work stress among nurses. Shared vision demonstrates a positive and significant association with self-reported quality of care (*β* = 0.195, *P* < 0.05). However, it does not show statistically significant associations with organizational commitment, self-directed learning, or work stress. Open-mindedness was only significantly and positively associated with self-directed learning (*β* = 0.169, *P* < 0.10). Overall, the results suggest that different dimensions of organizational learning (commitment to learn, shared vision, and open-mindedness) have varying impacts on nurse characteristics in CHCs.

## 4. Discussion

Our research findings revealed that 38 urban Shanghai, Shenzhen, Tianjin, and Jinan CHCs demonstrated a high level of organizational learning, with a particular emphasis on commitment to learn, followed by shared vision and open-mindedness. Nurse managers in New South Wales, Australia, stroke units also reported a high level of organizational learning culture [31]. In our study, strong organizational learning within these CHCs emerged as a significant predictor of nurses' self-directed learning and the quality of care they reported. There was no observed correlation between this organizational learning and nurses' levels of organizational commitment or work stress.

Organizational learning is viewed as a dynamic knowledge-based process, involving a fluid transition between various levels of action, spanning from individual to group, and further to the organizational level, and vice versa [32]. Therefore, the investigation into the relationship between organizational-level organizational learning and individual-level self-directed learning is of paramount importance. The previous research study has indeed examined various relationships between different variables and self-directed learning, including factors such as self-efficacy, organizational support, performance, and problem-solving ability [33–35]. However, there has been comparatively less emphasis on investigating the connection between organizational learning and nurses' self-directed learning.

Although not specifically focused on the nurse population, the four studies collectively offer valuable insights into the practices and impacts of organizational learning and self-directed learning across diverse contexts, yielding results consistent with this study: the first study explored the implementation of Senge's five disciplines of learning organization within an innovative motorcycle manufacturing company in Indonesia. It demonstrated that integrating these disciplines into daily work-life fosters self-directed learning among employees, emphasizing the importance of shared vision, personal mastery, team learning, and systems thinking [36]. The second study, involving a survey of 175 members of Civil Defence Emergency Preparedness Teams in South Korea, highlighted the positive influence of organizational learning on members' self-directed learning [37]. In the third study, which included 745 participants from 20 private universities, findings indicated a direct and positive relationship between organizational learning culture and self-directed learning [38]. Lastly, the fourth study focused on 521 public librarians in Seoul, Incheon, and Gyeonggi-Do, examining the impact of organizational learning readiness on their perceived self-directed learning ability. The study proposed strategies for enhancing organizational learning readiness to improve self-directed learning outcomes [39]. These studies collectively support the result of this study, emphasizing the significant role of organizational learning practices in promoting self-directed learning across various professional settings.

We defined self-directed learning as the ongoing enhancement of personal clinical expertise and medical knowledge. This underscores the critical importance of fostering a culture of organizational learning within CHCs. Our study found that nurses working in CHCs with a high level of organizational learning, particularly in terms of commitment to learn, were more likely to report elevated levels of self-directed learning. This observation highlights the potential link between organizational learning culture and nurses' ability to take charge of their own learning and professional development. This research study can illuminate the mechanisms through which nurses can leverage their organization's knowledge resources to enhance their personal learning and professional development.

In a study conducted in 2022 among healthcare department employees in Pakistan, it was discovered that the presence of an organizational learning culture exerted a positive and substantial influence on employee performance [40]. Learning organizations provide nurses with the opportunity to enhance the feedback mechanism for staff performance [41]. Furthermore, the concept of organizational learning has long been recognized as a pivotal factor determining an organization's sustained performance [19]. The relationship between healthcare quality and performance is well-established [42], prompting an exploration of whether organizational learning can impact healthcare quality from a novel standpoint. While extensive research has delved into the factors influencing healthcare quality [43, 44], there remains a conspicuous dearth of comprehensive information concerning the connection between organizational learning and healthcare quality.

Although the literature review did not find exact evidence of the relationship between organizational learning and care quality, we found several studies on the correlation between organizational learning and patient safety. Patient safety culture is crucial within healthcare systems as it significantly impacts the quality of care provided. A study involving 101 oncology nurses from two large Saudi tertiary care hospitals found that organizational learning positively influences patient safety culture [45]. Another study aimed to examine safety culture among registered nurses in four tertiary care hospitals in Thailand. Findings revealed that continuous improvement in organizational learning positively impacts safety culture [46]. Recent research indicates that a hospital has effectively enhanced nurses' theoretical knowledge and stabilized their practical skills. This was achieved by utilizing a nurse training system grounded in the principles of a learning organization. The system encompasses strategies such as comprehensive training, continuous management, and team learning. Consequently, the hospital has seen a reduction in the incidence of adverse nursing events [47]. Overall, these studies highlight the importance of promoting organizational learning for patient safety in healthcare settings. Our study revealed that nurses operating within CHCs characterized by a high level of organizational learning, especially in terms of commitment to learn and shared vision, are more inclined to report a high level of quality care. Consequently, this study offers a fresh perspective on enhancing the quality of care provided by nurses through the establishment of a learning organization.

Our study also introduced a novel perspective regarding the relationship between nurses' organizational commitment and organizational learning. Prior research has frequently indicated that organizational learning or specific dimensions encompassed within organizational learning significantly impact organizational commitment. For example, Yafang found a notable positive correlation between the presence of a “learning organization” and organizational commitment [48]. Darban et al. found that shared vision allowed nurses to align their activities with the organization's goals and direction, thereby enhancing their commitment to the organization [49]. On the other hand, studies conducted in China focusing on hospital nurses yielded the following findings: one demonstrated that training had a positive impact on organizational commitment, while another study revealed a positive correlation between nurses' job satisfaction, psychological empowerment, and organizational commitment [50, 51]. While there may be differences between hospital environments and primary healthcare environments, potentially leading to varying research outcomes, these two studies provide us with a hint that, at present, nurses may be more influenced by fundamental working conditions and resources, such as training opportunities, job satisfaction, and psychological empowerment, rather than organizational learning, regarding organizational commitment. In conclusion, the absence of a significant impact of organizational learning on the organizational commitment of primary care nurses represents a complex outcome. To gain a deeper understanding of this issue, further research is necessary to explore the interplay of these factors.

While the previous research study has predominantly focused on identifying factors associated with work stress, including but not limited to emotional exhaustion, depersonalization, personal accomplishment [52], working hours per week, and anxiety [53], most studies have concentrated on understanding which elements or stressors have negative effects on individuals. Not all stressors have adverse effects on individuals or organizations, despite increasing strains or demands. To yield positive outcomes, managers should aim to eliminate hindrance stressors while concurrently introducing challenge stressors within the workplace [54]. Our study represents a relatively novel exploration into the relationship between organizational learning and work stress. We conceptualized work stress as a set of stressors that positively contribute to the enhancement of nurses' skills and career development. Our study findings indicate that, aside from the “commitment to learn” dimension, the organizational learning and the other two dimensions had no statistically significant impact on nurses' work stress. The lack of a significant influence of organizational learning on nurses' work stress in China's primary healthcare settings may be attributed to various factors. These factors could include cultural differences, organizational support, or specific job demands. However, this study underscores the importance of continued exploration of organizational learning's role in enhancing nurses' career development and the quality of care within the evolving landscape of China's primary care system.

In addition to the aforementioned interpretation within the context of the wider literature, the findings of this study are also of significance to other regions and globally. Research on “learning organizations” within the health sector is predominantly focused on high-income countries and hospital settings, with only limited studies examining primary healthcare facilities, especially in low-and middle-income countries [55]. This study, targeting CHCs in China, presents an empirical case illustrating a potential pathway to enhance the quality of care in resource-limited environments through improved organizational learning levels. It offers a model that can be replicated by countries with similar contexts.

Countries with similar healthcare constraints, such as resource limitations and high patient loads—common in regions including Cameroon where nurses face significant emotional strain due to heavy patient burdens—can benefit from the insights gained through this study [56, 57]. Specifically, the implementation of structured learning environments and continuous feedback mechanisms can be adapted to local contexts. Moreover, in other regions, it could further validate the effectiveness of organizational learning frameworks in different cultural or healthcare settings.

The benefits of adopting learning organization frameworks extend beyond developing countries. For example, a study in Canada found that such frameworks positively impact daily nursing tasks [58], corroborating the findings of this study that emphasize the importance of career development and supportive learning environments in enhancing medical service quality. These findings underscore the universal applicability of learning organizations in both developing and developed settings.

Since 2012, the United Nations General Assembly has called on governments worldwide to advance towards Universal Health Coverage (UHC). A pivotal recommendation for UHC is that countries engage in ongoing learning [55]. Research studies have confirmed that adopting learning organizations is recognized as a strategic approach to improve knowledge management and promote continuous professional growth within healthcare sectors globally [58]. Therefore, the results of this study should encourage global health policymakers to intensify their focus on and explore effective strategies for fostering an organizational learning culture, which could substantially contribute to achieve universal health coverage.

### 4.1. Strengths and Limitations

The following limitations should be noted when interpreting our findings: First, this study is cross-sectional, confirming associations between CHCs' organizational learning and nurse-level variables rather than establishing causation. Our research team is preparing to conduct in-depth longitudinal investigations focusing on organizational learning in CHCs in the next few years to explore the causal relationship between organizational learning and nurse variables. Second, we recruited 175 nurses from 38 CHCs in four large cities. The primary care organization in these cities, including infrastructure building and primary care professionals, is relatively well established. While our results provide valuable information for further primary care reform in these well-developed cities in China, they may not necessarily be generalizable to other primary care settings. Future research should explore the generalizability of our findings across diverse healthcare systems and contexts. Third, CHCs selection was based on convenience rather than randomness. However, during sampling, we considered variations in practice size, patient volumes, and CHC ownership in each city to enhance the relative representativeness of CHCs and nurses. This approach may have potentially compromised the representativeness of the sample and introduced additional biases. Future research endeavours should consider employing more rigorous sampling techniques to enhance the reliability of the findings. Fourth, despite implementing quality control measures, reliance on self-reported data may introduce response bias and subjectivity. Future research should develop context-based primary care quality indicators for objective data collection.

While we have acknowledged several limitations to this study, our research significantly narrows the understanding gap regarding organizational learning within primary care, especially in developing countries. It establishes a notable association between CHCs' organizational learning and primary care nurses. This study offers a fresh perspective on enhancing nurses' personal self-directed learning and improving the quality of care provided by nurses through the establishment of a learning organization. It identifies promising areas for further investigation, making a relevant contribution to the fields of primary care and healthcare quality improvement.

## 5. Conclusion

This research examined 38 urban CHCs, revealing a strong level of organizational learning, with a focus on commitment to learning, shared vision, and open-mindedness. This study also explores the relationship between organizational learning and the well-being and work performance of primary care nurses, broadening our understanding of the role of organizational learning in primary care by highlighting its correlation with nurses' self-directed learning and the quality of care they provide. In conclusion, the positive correlation uncovered in this study suggests that strategic management reforms should focus on promoting a learning-oriented culture that can significantly improve nurses' self-directed learning capabilities and the quality of care provided.

The experience and results from this study, along with a thorough examination of the limitations inherent in various study designs, also could guide further, more refined research efforts. These further studies may examine whether there is a causal relationship between the level of organizational learning and nurse-level variables (such as self-directed learning and quality of care) in urban CHCs in China. In addition, they may also test whether the organizational learning level of primary care institutions in rural areas of China and its relationship with nurse-level variables exist. Both can help establish a solid foundation for further development of learning organizations.

### 5.1. Implications for Nursing Management

As China advances towards establishing a high-quality primary care system, the role of primary care nurses becomes increasingly crucial in addressing the challenges posed by the shortage of primary care physicians. It is essential that policies focus not only on structural inputs but also on reforming internal management practices. This study highlights the importance of establishing a culture of organizational learning, which is vital for effective nursing management in primary care settings within China.

First, evaluating organizational learning in 38 CHCs provides managers with a clear understanding of the current level of learning within the organization. This understanding enables them to develop targeted strategies for enhancement based on specific needs. To promote learning effectively, it is advisable to offer nurses' tailored learning plans and career development opportunities, such as regular training and team building activities.

Moreover, the presence of sustainable feedback learning loops relies on shared organizational visions, and the creation of these shared visions requires active participation [59]. To effectively promote organizational learning, nursing managers must recognize and empower nurses to drive these initiatives being critical, as their active engagement is necessary for success; they should concentrate on fostering shared visions that align with evolving healthcare needs; at the same time, managers should ensure that each nurse can get timely and constructive learning feedback and provide necessary support.

Third, scientific and medical advancements present a significant opportunity for healthcare institutions to embrace a learning organization culture [55]. According to a McKinsey report, artificial intelligence can significantly enhance the speed and accuracy of diagnostics, allowing practitioners to access more knowledge more rapidly and easily. This technology is poised to transform medical education by shifting the focus from rote memorization to innovation, entrepreneurship, continuous learning, and interdisciplinary work. To achieve this, all frontline workers need to integrate AI into their workflows [60]. Managers can leverage this approach to improve knowledge acquisition among nurses, thereby enhancing their learning abilities, anticipating advancements in medical knowledge that can improve nursing practices, and elevating the quality of patient care provided by nurses.

## Figures and Tables

**Table 1:** Organization-level variables and measurement items.

Variables	First-order factors	Measurement items
Organizational learning	Commitment to learning	Managers basically agree that our organization's ability to learn is the key to our competitive advantage
		The basic values of this organization include learning as a key to improvement
		The sense around here is that employee learning is an investment, not an expense
		Learning in my organization is seen as a key commodity necessary to guarantee organizational survival
		The organizational culture of our organization does not regard employee learning as a top priority^a^
		“Once we stop learning, the future will be in danger” is the consensus of this organization
	Open-mindedness	We have a clearly defined concept of the organization's positioning and future development
		Each department and section of the organization has a common organizational vision
		All employees of the organization are committed to the achievement of organizational goals
		Heads of departments and departments in the organization share their vision with their subordinates
		The organization does not have a clear and articulated vision^a^
		The employees of the organization feel that they are responsible for the future direction of the organization
	Shared vision	The organization is not afraid of us to question its various assumptions about operation and management
		Heads of departments and sections do not like to have their views questioned^a^
		The organization considers it important to be inclusive of diverse voices
		Our employees are encouraged to think beyond the norm and creatively
		The organizational culture of the organization does not emphasize continuous innovation^a^
		This organization places great importance on originality

*Note*. Items were measured using 5-point scales (1 = strongly disagree and 5 = strongly agree). ^a^Reverse-coded item.

**Table 2:** Nurse-level variables and measurement items.

Variables	Response categories	Measurement items
Self-directed learning	Likert 5-point scale 1 = strongly disagree and 5 = Strongly agree	You actively participate in all kinds of training and strive to improve the level of business and the quality of care
		You use your spare time to enrich your medical knowledge and improve your professional level
		You consciously and humbly learn from other highly skilled medical professionals

Quality of care	Likert 4-point scale	How do you assess the quality of care you provide to patients within this institution?
	1 = not good	
	2 = moderate	
	3 = good	
	4 = excellent	

Organizational commitment	Likert 5-point scale 1 = strongly disagree 5 = strongly agree	I have deep affection for the people and events of this organization
		I would be very happy to spend the rest of my career with this organization
		I feel obligated to remain with the organization
		I really feel as if this organization's problems are my own
		This organization has a great deal of personal meaning for me
		I do feel a strong sense of belonging to my organization

Work stress	Likert 5-point scale 1 = Strongly disagree 5 = Strongly agree	The number of projects and tasks I must complete at work is large
		The volume of work that I must accomplish in the allotted time is heavy

**Table 3:** Description of nurses and organizational characteristics.

Characteristics	Mean ± SD (range) or *n* (%) (units)
Nurse level (*n* = 175)	
Age	34.93 ± 7.79 (22–61) (years)
Sex	
Male	3 (1.71)
Female	172 (98.29)
Education level	
High school or below	12 (6.90)
Junior college/college	163 (93.10)
Years of working experience	11 ± 8.56 (0.2–40) (years)
Organizational level (*n* = 38)	
Organizational ownership	
Government	23 (60.53)
Public hospital	15 (39.47)
Accredited	
No	16 (42.11)
Yes	22 (57.89)
Organizational size	
≤35	13 (34.21) (employees)
36–55	7 (18.42) (employees)
56–100	7 (18.42) (employees)
>100	11 (28.95) (employees)

*Note*. The nurse data nested within 38 CHCs. Organizational ownership: government-managed CHCs or hospital-managed CHCs. Accredited: accredited by national or provincial health authorities or not. Organizational size: number of all staff.

**Table 4:** Main variables, Cronbach's alpha, and questionnaire score.

Variables	Item numbers	Cronbach's alpha	Mean	SD
Organizational learning	18	0.88	4.09	0.52
Commitment learning	6	0.74	4.25	0.60
Shared vision	6	0.81	4.07	0.59
Open-mindedness	6	0.72	3.96	0.61
Self-directed learning	3	0.83	4.16	0.72
Quality of care	1	—	3.17	0.54
Organizational commitment	6	0.94	4.04	0.88
Work stress	2	0.85	3.39	0.96

*Note*. SD: standard deviation.

**Table 5:** Correlation matrix of the main variables.

Variables	Organizational learning	Self-directed learning	Quality of care	Organizational commitment
Organizational learning				
Self-directed learning	0.179^*∗∗*^			
Quality of care	0.145^*∗*^	0.272^*∗∗∗*^		
Organizational commitment	0.149^*∗∗*^	0.507^*∗∗∗*^	0.266^*∗∗∗*^	
Work stress	−0.105	0.018	0.076	−0.154^*∗∗*^

*Note*. ^*∗*^*P* < 0.10; ^*∗∗*^*P* < 0.05; ^*∗∗∗*^*P* < 0.001.

**Table 6:** Multilevel linear models examining the association between organizational learning and nurses' variables.

Parameters	Self-directed learning			Quality of care			Organizational commitment			Work stress		
	*β*	SE	*P* value	*β*	SE	*P* value	*β*	SE	*P* value	*β*	SE	*P* value
Intercept	2.489^*∗∗∗*^	0.707	<0.001	2.194^*∗∗∗*^	0.532	<0.001	1.951^*∗∗*^	0.969	0.044	4.066^*∗∗∗*^	0.975	<0.001
Key organizational variable												
Organizational learning	0.272^*∗∗*^	0.119	0.022	0.234^*∗∗*^	0.087	0.007	0.265	0.183	0.146	−0.226	0.167	0.175
Control variables												
Nurse level												
Age	0.001	0.013	0.971	−0.001	0.01	0.921	0.008	0.015	0.578	0.002	0.017	0.909
Sex (ref = male)												
Female	0.164	0.397	0.680	−0.147	0.308	0.632	0.278	0.466	0.551	0.183	0.539	0.734
Education level (ref = high school or blow)												
Junior college/college	0.280	0.208	0.177	0.150	0.161	0.349	0.135	0.247	0.585	−0.043	0.283	0.879
Years of working experience	0.009	0.011	0.381	−0.001	0.009	0.960	0.011	0.014	0.419	0.017	0.016	0.265
Organizational level												
Organizational ownership (ref = government)												
Public hospital	−0.068	0.162	0.676	−0.054	0.115	0.640	0.016	0.262	0.950	−0.294	0.231	0.201
Accredited (ref = no)												
Yes	−0.105	0.127	0.407	0.011	0.092	0.906	−0.099	0.198	0.617	0.139	0.181	0.443
Organizational size (ref = ≤35)												
36–55	0.366^*∗∗*^	0.177	0.039	0.110	0.128	0.390	0.339	0.277	0.221	0.234	0.248	0.347
56–100	0.022	0.203	0.913	0.221	0.145	0.129	0.449	0.326	0.169	−0.337	0.286	0.239
>100	0.104	0.187	0.578	0.041	0.134	0.759	0.194	0.301	0.519	−0.326	0.267	0.222
ICC	0.123			0.060			0.219			0.094		
AIC	(377.013) 375.851			(290.889) 287.867			(444.730) 448.611			(477.673) 480.625		

*Note*. *β*: coefficient; SE: standard error; ICC: intraclass correlation coefficient; AIC: Akaike Information Criteria. AIC values in parentheses represent the AIC of the null model. ^*∗∗*^*P* < 0.05; ^*∗∗∗*^*P* < 0.001.

**Table 7:** Multilevel linear models examining the association between organizational learning's three first-order factors and nurses' variables.

Three first-order factors of organizational learning	Self-directed learning			Quality of care			Organizational commitment			Work stress		
	*β*	SE	*P* value	*β*	SE	*P* value	*β*	SE	*P* value	*β*	SE	*P* value
Commitment to learning	0.229^*∗∗*^	0.098	0.020	0.178^*∗∗*^	0.070	0.011	0.207	0.157	0.188	−0.326^*∗∗*^	0.123	0.008
Shared vision	0.177	0.109	0.105	0.195^*∗∗*^	0.078	0.012	0.262	0.161	0.103	−0.043	0.153	0.780
Open-mindedness	0.169^*∗*^	0.099	0.089	*0.119*	*0.075*	0.110	0.122	0.153	0.425	−0.121	0.139	0.388

*Note*. The control variables remain the same as in Table 6. ^*∗*^*P* < 0.10; ^*∗∗*^*P* < 0.05.

## Data Availability

Data are available upon request due to privacy/ethical restrictions. The data will be shared on reasonable request to the corresponding author.
